# Fetal Body Imaging: When is MRI Indicated?

**DOI:** 10.5334/jbr-btr.1378

**Published:** 2017-11-18

**Authors:** Marie Cassart

**Affiliations:** 1 Hôpitaux Iris et Iris Sud, BE

**Keywords:** Fetal MRI, prenatal diagnosis, thoracoabdominal fetal anomalies

## Abstract

Recent improvements in fetal therapies and perinatal care and the multidisciplinary involvement in fetal medicine have increased the demand for more accurate prenatal diagnosis. Fetal Magnetic Resonance Imaging (MRI) is a complementary imaging technique for the assessment of thoraco-abdominal anomalies for which Ultrasonography (US) is not conclusive. It is indicated in selected situations to precise the prognosis in diaphragmatic hernia, to characterise the nature and extension of a pulmonary malformation, to locate a bowel atresia or to better depict an abdominal cystic lesion or tumoural extension. It has become a mandatory complementary diagnostic tool and improves the management of the fetuses and newborns.

## Introduction

Imaging modalities are essential in fetal medicine. The routine follow-up of fetuses is achieved by Ultrasonography (US), which is and remains the first level screening technique.

Recent improvements in fetal therapies (fetoscopic surgery) and in perinatal care have increased the demand for more accurate prenatal diagnosis. Therefore, complementary imaging is mandatory in various clinical situations either to clarify an US diagnosis or to precise the anatomy and extension of a lesion. Fetal magnetic resonance imaging (fMRI) is performed in selected indications following US [1]. Undoubtedly, the first area of investigations for fMRI was the central nervous system. Its use has been significantly widened to thoraco-abdominal indications in case of pulmonary or digestive tract malformations, for instance for characterizing cystic or solid masses [2,3,4].

In the following pages, we will precise in which fetal thoraco-abdominal anomalies MRI has an additional value in prenatal diagnosis.

## Fetal Chest Anomalies

Congenital chest malformations range from small and asymptomatic entities to large space occupying masses that require immediate neonatal respiratory support and surgical treatment. Obviously, US enables a first detection of the lesion and pejorative signs when applicable. fMRI thanks to its multiplanar capacity, high anatomical definition and optimal tissue characterisation is a useful complementary imaging tool.

### Congenital diaphragmatic hernia (CDH)

**Figure 1:**
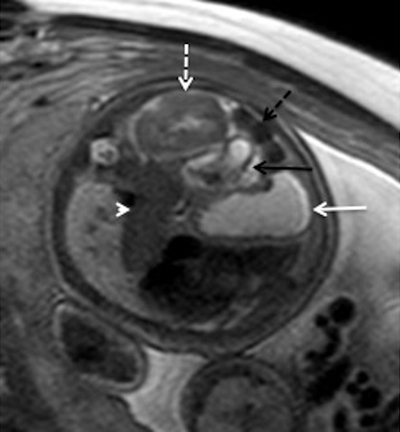
Left diaphragmatic hernia content. Axial T2-weighted sequence on a 33 week-old foetus with left diaphragmatic hernia. The content of the hernia is well characterized showing the stomach (white arrow), the spleen (arrow head), the kidney (dotted arrow), the small bowel (black arrow) and the colon filed with meconium (dotted black arrow).

CDH concerns one out of 4000 live births. US allows to establish the diagnosis but recent care advances (including maternal steroid therapy, fetal tracheal occlusion) require more accurate information regarding the severity of the malformation. The prognosis of such a malformation when isolated relies on its side and location (intra-pleural or not), time of discovery, precise content (position of the liver) and residual lung volume. In this context, fMRI is a good complementary tool that helps in better characterizing the anomaly [5]. Thanks to its high contrast resolution, fMRI differentiates more easily between intra pleural hernias from hiatal hernias or simple diaphragmatic eventration. It better defines the content of the hernia (Figure 1) and the volume of liver herniated in the chest which is considered as an independent negative predictor of perinatal outcome [6]. The residual lung volume can be evaluated by planimetry on T2-weighted sequences and is useful in predicting the respiratory function at birth. Those parameters are of great interest in predicting survival [7].

### Congenital lung malformations (CLM)

CLM include congenital cystic adenomatoid malformation, sequestration, bronchial atresia and bronchogenic cyst. It encompasses a large spectrum of lesions generated by airway obstruction in utero. The type of malformation depends on the timing of the obstruction, its level and severity. There is a considerable debate surrounding the classification of these lung lesions currently defined as congenital pulmonary airway malformations (CPAM) representing a wide continuum of lung anomalies. Additionally, the lesions are often mixed rendering the prenatal diagnosis difficult.

#### Congenital cystic adenomatoïd malformations (CCAM)

CCAM is the most common congenital lung malformation and accounts for 30 to 40% of all congenital diseases. They correspond to abnormal cystic lung tissue connected to the bronchial tree. They are classified (Stocker’s classification) according to the size of the cysts. These lesions are easily diagnosed by US because of their typical hyperechoic pattern including cysts. The role of fMRI is first to evaluate their volume compared to the normal lung [8], second to define as precisely as possible the affected pulmonary lobes thanks to the hypersignal pattern of the malformations. This improved evaluation of their extension may render useless the indication for a neonatal thoracic CT scanner. fMRI may also help to differentiate pulmonary malformation from huge cystic thoracic masses.

#### Bronchopulmonary sequestration (BPS)

**Figure 2:**
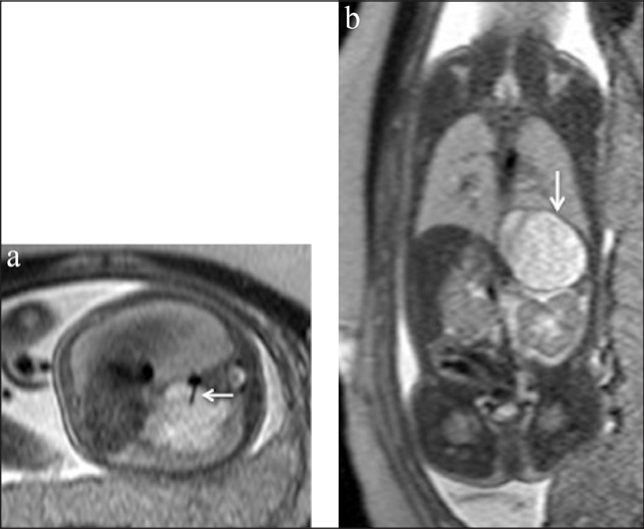
Intra diaphragmatic sequestration. Axial **(a)** and coronal **(b)** T2-weighted sequences on the thorax of a 27 week-old foetus showing a well circumscribed hyperintense lesion with a systemic feeding vessel (a, arrow) confirming the diagnosis of a sequestration. The hypointense capsule (b, arrow) is suggestive of an intradiaphragmatic lesion that was confirmed by surgery.

BPS is a developmental lung malformation that accounts for 6% of congenital thoracic lesions. It has a systemic arterial supply and is disconnected from the bronchial tree [9]. The detection of the systemic arterial vessel characterizing the sequestration may be better visualised on fMRI as well as the topography of the lesion which can be supra, intra or infra diaphragmatic [10] (Figure 2). This anatomical information is very important for the diagnosis and the surgical approach that can be planned before birth.

#### Bronchial atresia (BA)

BA sequence arises from interruption of the development of the airways and can occur at any level of the bronchial tree. It is hypothesized that BA results from an ischemic insult. On US, it appears like a non-specific hyperechogenic distended lung. fMRI may help in depicting the normal bronchovascular dichotomisation and the central branching mucocele appearing hypersignal on T2-weighted sequences. In cases affecting the upper lobes, an extrinsic compression can be better delineated by fMRI.

#### Bronchogenic cyst (BC)

BC is a distended blind bronchus resulting from abnormal budding of the ventral foregut. It is typically developed in the mediastinum (near the carina) less commonly it can develop in the lung parenchyma. It can be compressive on the mediastinum and airways leading to obstructive distal pulmonary distension. It usually appears like a mediastinal simple cyst. A differential diagnosis with other cystic lesions (lymphangiomas, cystic teratomas…) has to me made for more complex lesions (haemorrhagic content). fMRI helps in depicting the lesion itself and the associated lung anomalies (distended lung with increased T2 hypersignal) due to bronchial compression.

To summarize, in foetuses affected by CLM, fMRI may precise the diagnosis (according to their aspects and vascularisation), precise their extension and consequently differentiate between lesions that are more likely to cause fetal or neonatal distress requiring urgent surgical intervention. However, the overall rate of characterization is low because of a high percentage of mixed lesions and frequent non-specific imaging findings [11,12].

## Fetal Abdominal Anomalies

Abdominal anomalies concern the digestive, the urinary or the genital tract and sometimes malformations involving both systems like in urogenital sinus or cloacal dysgenesis. Abdominal tumours are not rare during fetal life. Again, the role of fMRI is to better characterize these lesions and precise their extension and impact on the development of adjacent organs.

### The digestive tract

#### Esophageal atresia (EA)

**Figure 3:**
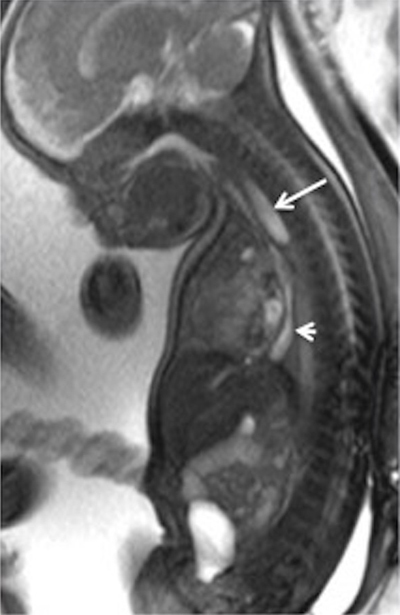
Oesophageal atresia. Sagittal T2-weighted sequence on the midline of a foetus referred for hydramnios. The fMRI demonstrates the proximal blind pouch (arrow) and the long distal oesophagus (arrow head). This examination allows predicting an end to end surgical anastomosis.

EA results from an incomplete division of the foregut into the airways anteriorly and the digestive tract posteriorly. It is encountered in 1 out of 3500 live births. The sensitivity of prenatal diagnosis is still quite poor (40%). Various types are described and the most common type is the type III with a blind proximal pouch and a distal fistula between the airways and the distal esophagus leading to indirect signs (presence of a small stomach, discrete polyhydramnios). The visualisation of the blind proximal pouch, which is pathognomonic, may be difficult. fMRI, thanks to the high contrast resolution of T2-weighted sequences and dynamic acquisitions focused on the midline may help to establish the diagnosis by the visualisation of the distended proximal pouch when the fetus swallows and even to precise the length of the gap between the proximal and distal part of the oesophagus [13,14] which is an important information in terms of surgical procedure (end to end anastomosis versus colonic interposition) (Figure 3).

#### Bowel atresia

**Figure 4:**
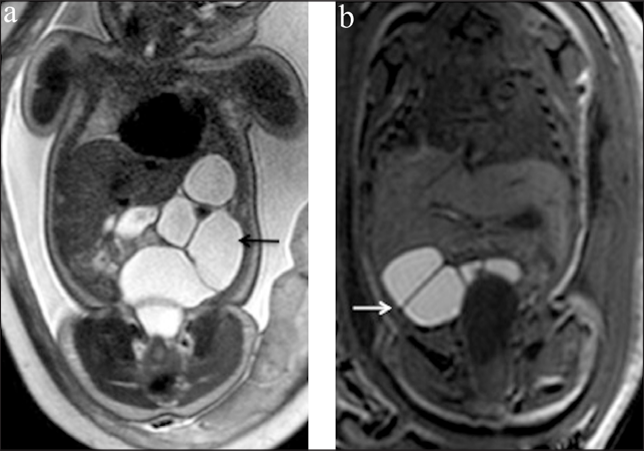
Differentiation between proximal and distal small bowel occlusion by bowel signal content. **(a)** Coronal T2-weighted sequence on the abdomen of a 32 week-old fetus with distended hyperintense loops (arrow) in the left hypochondrium corresponding to dilated proximal small bowel filled with ingested amniotic fluid in a context of proximal small bowel atresia. **(b)** Coronal T1-weighted sequence on the abdomen of a 37 week-old fetus with distended hyperintense loops (arrow) in the right flank corresponding to dilated distal small bowel filled with meconium in a context of ileal atresia.

Digestive tract atresia occurs in 1 out of 4000 births, it is thought to result from ischemic insult [15]. It appears on US as distended loops either filled with translucent amniotic fluid in proximal cases or more echogenic content in distal occlusions. It is important to know that small bowel atresia is much more common than colonic atresia. fMRI thanks to the spontaneous contrast resolution of the bowel content (amniotic fluid hyperintense on T2 and meconial content hyperintense on T1) (Figure 4) may help to identify the level of the occlusion and/or diagnose a microcolon [16]. More than that, fMRI better than US may depict meconial pseudo cysts, resulting from perforation, thanks to the typical meconial signal of their content [17,18].

### Pelvic cystic lesions

Pelvic cystic lesions are frequently encountered during fetal life. They may correspond to duplication cysts, ovarian cysts in female fetuses, ano-rectal malformations or genito-urinary sinus… and the list is not comprehensive. In cases depicted by US, fMRI may help for the differential diagnosis by a better characterisation of the cyst content and a better depiction of the digestive tract calibre and position [19].

#### Ano-rectal, cloacal malformations and uro-genital sinus

Abnormal progression of the uro-rectal septum leads to incomplete separation of the digestive from the uro-genital tracts. It occurs in 1 out of 2500 to 5000 live births. In some rare cases the urinary and genital tracts are both abnormal leading to various forms of urogenital sinus. In other situations, the malformation involves the digestive tract potentially leading to a simple ano-rectal malformation or a more complex cloacal dysgenesis. These anomalies must be differentiated because their prognosis is very different. fMRI is useful to clarify the anatomy of the malformation by differentiating the urinary from de digestive tract [2] or to visualize a solitary intermediate signal pelvic cyst corresponding to the cloacal pouch. fMRI also better than US locates the distal rectal end in case of ano-rectal malformation [20,21]. fMRI also has a prognostic role, because such malformations carry a very poor functional outcome and interruption of pregnancy can be proposed in complex cases [22].

#### Ovarian cysts

**Figure 5:**
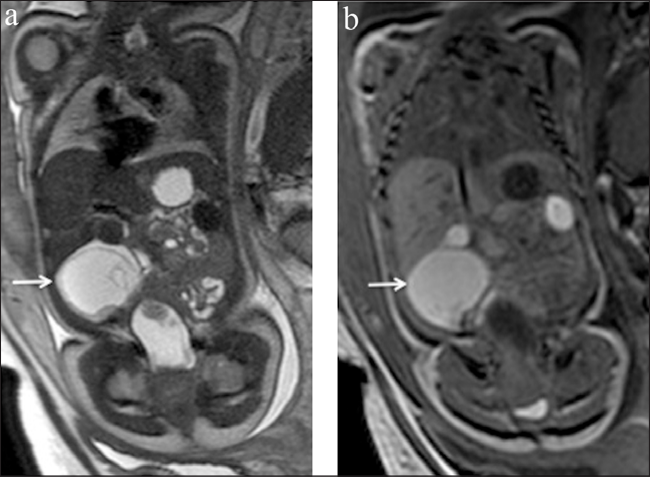
Characterisation of a pelvic cystic lesion-hemorrhagic ovarian cyst. **(a)** Coronal T2-weighted sequence on the abdomen of a 38 week-old female fetus presenting a hyperintense cystic lesion (arrow). **(b)** On the T1-weighted sequence slice performed at the same level, the lesion appears hyperintense. This T1 signal is highly suggestive of blood content leading to the diagnosis of a hemorrhagic ovarian cyst.

Ovarian cysts are the most common pelvic cystic masses encountered in female fetuses. They occur in 1 out of 2600 pregnancies. These cysts classically appear in the third trimester in relation with the hormonal environment associated with the gestation. Most appear as a unilocular thin walled cystic structure with anechoic content. Generally, no complementary imaging is requested but complications like bleeding or torsion can occur in utero leading to an increased size and modified content. In such complex cases, fMRI may help for the characterization of the cyst thanks to specific signal of its haemorrhagic content (Figure 5) [23].

### Abdominal tumours

#### Sacro coccygeal teratoma (SCT)

SCT is the most common location for teratoma in fetuses. The tumour arises from the anterior part of the coccyx and extends externally or inside the pelvis and abdomen. Most fetal teratomas are benign; consequently, their prognosis relies mainly on their intra pelvic/abdominal extension and spinal canal invasion [24]. Therefore, fMRI is a useful complementary tool to US to better define the topography of the lesion and its impact on adjacent organs [25]. fMRI is also mandatory to determine the cystic and solid component of the mass, as this has an important impact on the fetal prognosis and on the delivery management. Cystic parts can be drained before delivery to prevent dystocia whereas the tissular parts may be highly vascularized and responsible of blood shunting leading to cardiac failure and hydrops [26].

#### Lymphatic malformations (LM)

LM are composed of distended cystic lymphatic channels. They are mainly located in the cervico-facial and mediastinal regions but some can develop in the chest or abdomen. They appear as mainly cystic and septated masses. The cystic components appear anechoic but sometimes they display an echogenic content suggesting intracystic haemorrhage. These lesions typically infiltrate or displace the surrounding organs. Again, the main role of fMRI is to precise the extension of the lesion [27]. In thoracic locations, the main role of fMRI is to exclude respiratory tract infiltration or compression in order to prepare the obstetrical team for an EXIT procedure with perpartal intubation in case of trachea-bronchial occlusion. In abdominal locations, fMRI defines the extension and the impact on adjacent organs. This information is important considering the surgical procedure and prognosis of extended and infiltrative lesions.

### Urinary tract

#### Megabladder

In utero, a megabladder is defined in the second and third trimester as a bladder with a long axis on a midsagittal US scan of more than 30 and 50 mm respectively. Megabladders can be obstructive (urethral valve), secondary to reflux or dysplastic. The megabladder is an easy prenatal diagnosis but the etiology is sometimes difficult to establish in utero. fMRI may help in the visualisation of the urethra in case of thickened bladder wall with suspected urethral valve, it may also more easily than US exclude or confirm a microcolon in case of suspected megabladder, microcolon intestinal hypoperistalsis syndrome or cloacal dysgenesis (see above) [28]. In case of decreased amniotic fluid secondary to renal insufficiency, fMRI improves the visualisation of the kidneys and can evaluate lung volume to exclude hypoplasia, which significantly worsen the prognosis.

## Conclusion

fMRI is a complementary imaging technique for the assessment of thoraco-abdominal anomalies for which US is not conclusive. It helps to determine the nature of a lesion, extension of tumours, or precise the anatomy in case of complex malformations. It is an important support to diagnosis and consequently to prognosis. Therefore, it allows a better depiction of malformations and anomalies with a better comprehension by the multidisciplinary staff. Consequently, it has become a mandatory complementary imaging technique in selected indications and improves the prenatal and neonatal management of the fetuses and newborns.
